# That Backbone

**Published:** 1955-11

**Authors:** A. Gordon Heron


### THAT BACKBONE

### The Presidential Address, delivered on Wednesday, 13th October, 1954, at the Seventy-Sixth Session of the Bristol Medico-Chirurgical Society

BY

A. GORDON HERON, M.B., CH.B.

The Bristol Medico-Chirurgical Society is an old, distinguished and learned society, enhanced in its close association with the University and the Teaching Hospital, and I must thank you for accepting me as President for the coming year, because this is a very great honour indeed.

But I must confess, once and for all, that I feel quite inadequate, and entirely undeserving of this high honour which has been bestowed upon me. I can assure you, however, that I will serve the Society to the utmost of my ability.

And now, what have you got?

In trying to find out what I had to give you I have discovered that I am a G.P., and very little else. Also sad to admit, what our Secretary said has a bitter spice of truth, I am one of the oldest G.P.s in this area. There are still fortunately, a goodly number of genuine elders, wise, cultured, and supreme, from their growth in the basic calm of the early years of the century. But I, like many of the rest of you, was both fortunate and unfortunate in having dropped on my youthful shoulders, the mantle of the lost generation—those elder brothers of ours who went to war, and of whom, so pitifully few came back, or, if they did, were in many cases, too shocked and unsettled in their minds to undertake their intended vocations.

On 11th November, 1918, I was at school, attempting to combine the Classics with preliminary training as a machine-gun target. Two days later I was a first-year medical student at the University. On 29th February, 1924, I qualified. Next day I was locum house surgeon to Mr. Carwardine at Bristol Royal Infirmary. On 1st April, I was a house physician, and on 1st October resident obstetrical officer and "Gynae" house surgeon. In March, 1925, there was a pandemic of influenza, and my father asked if I could possibly be relieved to help him. On 17th March at 3 p.m. I was in the Greig-Smith Theatre, and at 4 p.m. I was a general practitioner in consultation with a physician over a case of status asthmaticus. Sometime in June I started on a year's introduction in general practice with that delightful man, Dr. Freddie Mayes—a year of very valuable and necessary education.

On 24th June, 1926, I got married, and on 12th July began practice on my own. And then there followed seemingly in rapid succession: getting started, getting going, the war, and the National Health Service. So that when I look back, and when Dr. Tryon said "why not talk about one of your hobbies?" I had little difficulty in coming to a conclusion that really I haven't any. I have plenty of interests but I am a "specialist" in nothing; just a general practitioner of a number of things. If there is one thing to which I have perhaps given a more than average amount of time and interest it is to medical politics, though whether to any useful purpose it would be difficult to say. So I have decided—not without a great deal of anxious thought—to stick to my last, to risk being very dull and commonplace, and to talk to you about "General Practice".

I have been encouraged to do this because several of you have expressed a hope that I would, and because I hope to be able to kindle in each one of you an active interest in some ideas—in this city of ours so sensibly indistinguishable from ideals!—for the positive health and efficiency of the Body Medical.

The title I have chosen for my ramblings is "That Backbone". If politics enter in I am unrepentant, because they will be the intrinsic politics of the profession, to which I doubt very much if we are giving sufficient thought—certainly not dynamic thought

—towards the efficiency and well-being of the profession as a whole, which means—and it is important always to remember this—the well-being of the patient, the whole patient—a matter which is the constant aim and object of good general practice.

Professor Geoffrey Jefferson has written "The Sick Person is *a whole* of incalculable diversity, and the future teaching and practice of medicine must be built as firmly upon this fact as upon the detailed complexity of scientific knowledge." That perhaps expresses my main theme.

I am going to look back for a while in order to view some of the trends that have led up to the present, and also to discuss some misapprehensions.

I grew up in an atmosphere of general practice. My father practised in Bristol for fifty-three years. My grandfather was a country practitioner in Northern Ireland, and for many years coroner for the south half of County Down. He had a very pleasant life as a doctor-farmer, but he worked steadily and lived thriftily till the age of eighty-three. My father began as an assistant in Totterdown, for three years, and then " put up his plate " in a developing area. After another five years he was able ' proudly ' to open a bank account—a year after I was born. Those were days of fierce, and sometimes bitter, competition, when a man was envied if he acquired appointment to a " club," a sort of benefit society, which paid anything from 2*s.* 6*d.* to 4*s.* 0*d.* per member, per annum, for which the Doctor undertook to provide attendance *and medicine*. For many years my father—and indeed it was the rule in general practice—had consulting hours from 9–10, 2–3 and 6–8, every day of the week, and at 9 a.m. on Sunday. There were no half-days nor free week-ends. Until he was forty-four he did all his visiting on a bicycle except for the occasional hire of a brougham during a winter epidemic.

In 1912 an inquiry was made by the Government into the earnings of general practitioners in six towns (selected by agreement with the British Medical Association). The books of 225 doctors were examined and the average income was £493 per annum, and represented every kind of medical service rendered, included every section of the local population, and worked out at 4*s.* 3*d.* per head. Although they could not see it at the time there is no gainsaying that the National Health Insurance Act was a godsend to general practitioners. For the first time they received a regular and not unreasonable payment for a class of patient from whom previously they had little, if anything at all. But wives and children were still to be precluded for another thirty-six years. It is interesting to note that at that time Dr. John Burns, President of the Local Government Board, was being pressed by his medical advisers to introduce a National Health Service linking up the public health work of local authorities with specialist and general medical practice.

But Mr. Burns hesitated and Mr. Lloyd George, that master of expediency, jumped in.

During that period round the turn of the century medical knowledge, which had begun a slow advance, gathered its ever-increasing momentum, resulting in a steadily growing requirement for hospital and specialist services. The Public Health medical services were initiated. It is interesting to note today that the first of these measures was "The Education (Provision of Meals) Act of 1906", and that the British Medical Association was active in pressing for the establishment of a school medical service. The Maternity and Child Welfare Act did not come until 1918. It has become the fashion in some quarters to state that these developments became necessary largely owing to the shortcomings of general practice. But how those poorly paid, single-handed, doctors could have been expected to go into action, unasked and free of charge, to deal with problems which, to begin with, were at best only dimly perceived, it is difficult to imagine. The truth is that the first Act was directly occasioned by the public concern over the so-called "C 3" population revealed by the medical examination of recruits for the South African war, and subsequent developments were stimulated by resuscitation of that concern, for the same reason, in the First World War. The maternity measures probably arose, partly owing to the same jolts to public expediency and conscience, and partly to the gradual relaxation of the false modesty of Victorian conventions, the falling birth-rate, and advance in knowledge. Convention decreed that the young married woman, when pregnant, passed into "an interesting condition", not to be revealed even to the family doctor until a few weeks before his attendance was likely to be required.

Playfair, a recognized authority in his time—he had a probe you will remember!—published his book on midwifery in 1898. In this book there are only two paragraphs concerning the maintenance of normal health in pregnancy, mainly directed to the control of constipation, and the value of proper corsets. There are also these two sentences—"It is often sufficiently easy to recognize a pelvic presentation by abdominal examination—*if we have occasion to make one* . . ." and again " . . . it rarely happens that deformities of the pelvis, except the gravest kind, are suspected before labour has actually commenced."

Advances in knowledge often germinate in curious ways. In 1900 Dr. Haig Ferguson of Edinburgh supervised the health of the inmates of a pre-maternity rescue home, and was so impressed with the normality of the girls' pregnancies and the fine condition of their babies, that he persuaded the Royal Maternity Hospital to offer similar supervision to their pregnant married women by means of an out-patients' clinic. The entrance to this clinic had to be arranged from an unfrequented side street, and great consideration given to the ladies' modesty in putting up an unobtrusive and discreetly worded direction-board.

J. W. Ballantyne, a colleague of Haig Ferguson, tookover the management of this antenatal clinic—the first in the country—and in 1901 *a bed* in the hospital was endowed for the study of diseases of pregnancy. Here then is one example of a simple beginning of a great social and medical advance, emanating solely from the clinical observation of an active mind. That development in this field was slow is not at all surprising considering the jungle of atavistic reaction that had to be cleared away. I am sure that a majority of general practitioners were not backward in trying to adopt these new ideas as soon as they became aware of them, but it cannot have been easy. Even in the earlier years of my own practice, my motives were impugned on several occasions by militant mothers, who made it quite obvious that unnecessary and uncalled for indecencies were being inflicted on their daughters. A fault which the general practitioner does have is, in fact, rather the opposite—an over-eagerness to venture upon a new idea, or method of treatment, without first giving it sufficient consideration or critical inquiry. This is, however, easy to understand because he has to live with his patients, he has to try to deliver the goods, he sees the failures and disasters; and he is all too conscious of the gaps in his armoury, and is constantly hoping to fill them. And it is worth remembering that in such ways a number of great advances have come from general practice.

Jenner recognized the relationship between smallpox and cowpox. Withering observed the diuretic effect of the foxglove leaf. Charles Turner Thackrah, a general practitioner in Leeds, became interested in the health of the working people, and in 1824 began intensive inquiries into the causes of disease and death among them. Perhaps the best illustration of the range of his work is depicted in the title of his book—*The Effects of the Principal Arts, Trades, and Professions, and of Civic States and Habits of Living, on Health and Longevity: and suggestions for the removal of many of the Agents which produce disease, and shorten the Duration of Life.* This book was published in 1831, and was the first comprehensive work of its kind in English. His untimely death, two years later, at the age of thirty-seven, has been described by Sir John Simon, as retarding the progress of industrial medicine for half a century. The careful observation and deductive reasoning of Bristol's own doctor, William Budd, established in pre-Pasteurian days the main facts about the spread of cholera and typhoid. And perhaps the greatest of all, was Dr. James Mackenzie, of Burnley, who after many years of careful and searching clinical observations, in the course of a very busy industrial practice, revolutionized the whole conception of heart disease. Had he not lived, I wonder should we still be condemning our missed beats and our innocent murmurs to bed for three months, and then to a life of invalidism, and letting others, without objective signs, die? Probably not. But it is salutary to remember that this greatest of doctors did not come to London until 1906, did not publish his book till 1908, and died only twenty-nine years ago. And to me, the most inspiring scene of all, is of Dr. Mackenzie, a self-made man if ever there was one, in 1918, at the age of sixty-five, slipping quietly away from Harley Street, abandoning the £8,000 a year consulting fees, to return to general practice; Why? Because he wanted to be able to study again the earliest symptoms of disease, because he wanted to be able to say to Mr. Smith and Mrs. Jones, "How do you *feel*?"

That these things are still possible can be seen in the work of Dr. W. N. Pickles, a country doctor in Wensleydale, who in the early thirties, by carefully collected observations established the fact that catarrhal jaundice is an infectious disease. Also, in anaesthetics, for at least two generations we have led the world, and until 1948, the great majority of anaesthetists were general practitioners.

In lesser things some of the usages and customs of general practitioners which have been the subject of magisterial rebuke or amusement have not always proved so wrong. I was taught that the diagnosis of gastritis for which general practitioners were so addicted to giving certificates, only revealed their ignorance, because such a condition did not exist—until the invention of the gastroscope. I was taught that the custom of my father and his contemporaries of giving two drachms of extract. Ergot. liq., post partum, and leaving another dose on the mantlepiece, was entirely useless because the liquid extract contained neither of the alcohol-soluble active principles. Some years later water-soluble Ergometrine was discovered, the most potent of them all. I was taught that the prescription of digitalis by General Practitioners for cases of cardiac weakness was indefensible—almost criminal—because digitalis could only be useful in auricular fibrillation. Only in recent years has it been proved scientifically that digitalis has a direct action on the myocardium. I am not decrying my teachers, I owe them too much. Just those were the accepted theories at that time.

But it does show that even in this modern world of science, the art of clinical observation is still of prime importance. It still happens that one year's scientific asseveration is sometimes contradicted with equal authority in the next.

It is perhaps significant that during the inter-war years, with the bright exception of Dr. William Pickles, standing almost alone, general practice produced little of distinction. And that may seem curious considering that it was during that period that general practitioners probably achieved their highest standing and financial reward.

That earnings, even then, though better comparative with preceding years, were still not generous, was shown in the statement of the Spens' Committee that according to their estimation general practitioners had been underpaid for an unknown number of years prior to 1939.

During the war years there was a definite diminution of income, and when the National Health Service came, those of us who were in rural or suburban practice were reduced still further.

The Danckwerts' award has brought the level up to something more like a living wage, There seems however to be an idea abroad amongst some members of the profession that Danckwerts has brought wealth to general practitioners. I regret that such is not the case. Do not forget that the general practitioner has to pay for everything except prescription and certificate pads, and that even the Treasury agreed two years ago that, on the average, 38·7 per cent. of his earnings are absorbed in practice expenses.

In my own partnership these expenses have risen by over 50 per cent. since 1948. So that I, for one, am still very glad of two or three small additional jobs, with the help of which I think I can just about break even with 1939, but I am having to work harder and longer to do so.

But it was during that inter-war period that the dislocation of the "Backbone" from the Body Medical began to make itself apparent, and was greatly and abruptly aggravated by the more rigid compartmenting of the various branches, introduced by the National Health Service, and the emphasis placed by the Act on the Hospital Service.

When I started in general practice it was still possible to continue most of the methods of investigation and treatment which had been learned in one's hospital training. It was only necessary, in the main, to seek help in cases of difficult diagnosis or where surgery was needed. It was possible, more or less, to keep up with advances, by contact with consultants, by reading, and by attending meetings of societies such as this. But the speed of progress accelerated greatly, and especially so during the war years when the opportunity and ability for keeping up were considerably diminished. (Also new specialities branched off.) So that the general practitioner developed something of an inferiority complex, and also found himself with the added anxiety of having to select the right specialist.

During the same time the kind of work done in hospital has diverged more and more from the kind of work done in general practice. Even when I began the change was discomfiting. From having been resident obstetric officer and acting senior resident officer, and consequently, lord of all I surveyed, and thus acquired an overweening self-confidence, I found myself trembling on patients' doorsteps. Could I recognize a case of measles and, if so, what to do about it? How to treat this impatient business man with influenza, and was I sure it was only influenza? How to advise that young mother on infant feeding? Whooping-cough—what did I know about whooping cough? Convulsions?—save us! Had this valuable child got tonsillitis or might it not be diphtheria? So that I was often very glad and grateful to be able to run home to Daddy—literally. Oh, what a fall was there! Today an embryo general practitioner, on leaving hospital, is a fish out of water indeed, and is bound to suffer accordingly. He knows little or nothing about general practice, and may never even have seen or spoken to a general practitioner, he feels cut off from much of what he had become capable, because many of his accustomed aids to diagnosis, and treatment techniques, are difficult—if not impossible—to carry out in the surgery or in the home, and kitchen-table operations are hardly justifiable under modern standards.

The trauma of this sudden severance may be considerable, and although he will achieve some recovery in discovering that there is much useful and interesting work to be done, and should find that he has attained a position of friendly esteem and regard, he may well continue to harbour feelings of frustration, of sterilization of acquired ability, of wastage of much of his elaborate training.

Contrary to some opinion, I do not believe that overworked word "status" comes into this picture. General practice did lose popular status in the early years of the Health Service but I think that, in the main, it has been regained.

The National Health Service found the hospitals understaffed at all levels, and still suffering severely from other post-war difficulties. Consequently for some years it became almost impossible to get patients admitted to hospital. And they were often discharged, or sometimes died, without any information being sent to the doctor. These things made people angry and distrustful. Hadn't Mr. Bevan made his irresponsible trumpeting of "come and get it?" *Ergo*—it must be there. Hadn't the general practitioners been the ones who had fought longest and loudest against the Health Service? They just weren't trying. It must be all their fault. But gradually over the years, things have improved. There is once more reasonable understanding and goodwill between registrars and housemen on the one hand and general practitioners on the other. And the Public have for the most part, discovered what they and their doctors are up against—that the Hospital Service cannot even now fulfil Mr. Bevan's turgid promises. So that if any section of the National Health Service has fallen short in the eyes of the public it is the consultant and hospital services.

In general practice the number of people who have chosen to revert to private practice must be infinitesimal. I myself can think of only one. Whereas, the number of those who began as private patients in July, 1948, and have since transferred to the Service, is quite considerable. On the other hand it would appear that an increasing number of people are, in effect, contracting out of the hospital service by joining one or other of the various provident associations scattered throughout the country, in an effort to insure private consultant and nursing home facilities for themselves and their families.

The biggest of these associations, the British United Provident, increased its membership by 9,700 in 1951, 24,700 in 1952, 36,000 in 1953. The Western Provident Association which is centred in Bristol, and is a comparatively recent foundation, had 370 new members in 1951, 630 in 1952, 1,570 in 1953, and already in the first six months of this year 1,360—more than seven every day. And these units represent families more often than single individuals. The annual premium varies from £3 5s. to £13. There is of course no income tax relief. A direct comparison of this state of affairs with that in general practice would not be fair because people will naturally tend to insure against the major disaster and the devil they don't know. Nevertheless this is scarcely the grand vision of the National Health Service. It is true that we have gradually achieved a certain degree of order in chaos, but it is still chaos. What it means is simply that the consultants with their registrars, house-officers, and other ancillary help, cannot cope in the hospitals with the needs of the populace. And the chaos does not rest there. It is in the profession itself. The Ministry, with a book of tables and slide-rule in front of them, and the Treasury behind, state that there are not enough consultant appointments for all the senior registrars, so that some of them must go. But if they go, who is going to do their work? Chaos will be still worse. And where are they to go to ? General practice? But general practitioners do not want to recruit from disappointed men, however brilliant they may be, compelled by artificial circumstances to leave their chosen path with its vista of a promised land, along which they have laboured so long—to leave this, for a job they do not want to do, and for which they are not equipped. And now, apparently, it is becoming difficult to recruit senior house-officers because the young men are seeing the red light further up the ladder.

A sorry state of affairs indeed. Consultants are, many of them, worried; registrars anxious and unhappy; prospective house-officers frightened away, and many of the younger general practitioners frustrated and discontented. The older ones have either resigned themselves to work out their time, or, if they feel for and believe in their vocation as I do, are irritated by its present state, and disturbed about its future prospect.

There is surely absolute justification for this general anxiety. Whereas if the present course of drift continues, the hospitals, when thay have shed their surplus registrars, may achieve some sort of *modus vivendi*, one cannot imagine that it will be for the better; certainly it will do nothing to help the case of the registrars, nor the separation of general practice, with the gradual deterioration that is almost bound to result.

It is necessary to consider what general practice is, and does, in order to decide how serious the situation may be, and how imperative the necessity for action, because there are members and, it would sometimes seem, sections, of the profession who have talked of general practice as being ineffectual, obscurantist, and of little account. This ill-considered outlook may have partly arisen from the fact that the average general practitioner, having started along his path, has followed it without thinking either of the importance, or the extent, of the work which he is doing, and so, even if he wished, is in no position to publish it from the house tops. Recently, however, general practice and the general practitioner have been examined by so many people from every possible angle that if we had not, long ago, lost the faculty, we should be suffering a continuous blush.

A number of interesting findings have emerged from these investigations. One of the first was that *eighty per cent.* of this country's illness is cared for by the general practitioner. That this represents mainly trivialities has been refuted. The *British Medical Journal* of 16th January this year contains a study by members of the Medical Research Council of a practice in north-west London. This practice was in a relatively poor area. Findings were that, of the possible patients, 72 per cent. consulted the doctor during the year; 36 per cent. of the consultations were on children under fourteen; only 12 per cent. of the children under four did not see the doctor during the year; 16 per cent. of all cases were of serious disease, and despite the proximity of hospitals, the work involved in treating these serious cases amounted to 43 per cent. of the total work of the practice.

With regard to the general practitioner himself, Dr. Stephen Hadfield, in his report on his investigations of 188 practices, states that 92 per cent. of the practitioners whose work he witnessed were adequate or better than adequate, and were undoubtedly interested and careful in the treatment of their patients.

"They give the necessary advice, supervise rehabilitation, prescribe carefully and do dressings and emergency treatment; and nearly all consider it their job to act as the patient's 'guide, philosopher, and friend', as well as giving him clinical care."

Of the doctors' premises, he found 10 per cent. to be unsuitable, but that these were usually in districts where the housing was bad: in one instance the doctor had to bolt his waiting-room chairs together to prevent their being taken away. Here is an example of the old adage that you can't make a silk purse out of a sow's ear, and such conditions must have accounted at least in part for the adverse findings of the Collings' Report. There, in brief, is a clearer picture than we have had before of the general practitioner and his premises.

What of his work? As one writer has put it, to define the practice of medicine as "extraordinary" and "ordinary" would express a much clearer differentiation than that of "specialist" and "general", practice.

The general practitioner should be able to undertake all ordinary medicine. He has often to be a father, friend, and confessor to his patients, and an adviser by request or self-appointment on almost everything under the sun. No-one else is in so advantageous a position to give advice.

As Professor McNee said in his Presidential Address in Glasgow—"every general practitioner is essentially a teacher, instructing his patients during illness and also, I would hope, on how to keep well." Particularly must he be ever mindful to try to combat fear. This factor of fear is probably more active, today, owing to ill-digested and indigestible publicity, than since the times of the evil eye. And it is easy, in one's own familiarity, to overlook the thinness of the popular veneer which, almost always overlies a fearful and abysmal ignorance. What Dr. Bluestone said, is true, that the moment a patient enters a hospital a new complication supervenes: fear.

Last, but perhaps most important of all: diagnosis. The general practitioner alone is so placed as to be able to detect the early signs of deviation from normal health. The responsibility of making the first diagnosis is a heavy one and demands knowledge, which may be insufficient, alertness and agility of mind—which may be blunted by fatigue or the common light of common hours—and the wisdom born of experience, so often the tired doctor's guardian angel. Prompt and accurate diagnosis is constantly becoming of greater importance with the availability of more precise weapons of treatment and increased powers of surgical technique, with less place remaining for reliance as much as formerly on *vis medicatrix naturae*. So that the general practitioner has to undertake a considerable load of responsibility, not only that which the patient places on his shoulders, and which he assumes on behalf of his patient, but of constantly endeavouring to keep pace with developments. That is not so easy for him as for doctors working in hospitals, especially when the whole of his work frequently entails a twelve-hour day and sometimes longer.

I have tried to discover any popular opinion on these matters, but there is obviously little interest in, or appreciation of, our difficulties. The English do not think—they only expect. But *The Economist* has pointed out that "it may be specialized medicine rather than socialised medicine that represents the graver danger." and says that "the narrow sectional interests which have been artificially stimulated by the National Health Service must now give way to a concerted effort." And *The Times* pronounces that "There can be no substitute for the able family doctor. He still holds in his hands the lives of his patients. No hospital or specialist service, however elaborate, can offset defective treatment in home or surgery. If General Practice is not restored to a new level of competence—some would say restored to its rightful place—the whole of British Medicine will suffer."

Is it possible to imagine that this "Backbone" can be dispensed with or superseded? If not, there must be a strong case for doing something about it. For many years there has been much talk round many tables; general practitioners have asked, committees have presented plans, and plans have been accepted, but in the main they lie on the table.

With regard to the Medical Curriculum I am not competent to enter into detail. Professor McNee said in Glasgow, "the limit has already been reached, even surpassed. It is longer than for any other profession, and the question arises of how much the average medical student must know, and learn, in his undergraduate career. To know all would require a lifetime." And again: "Academic theory of disease, detailed chemistry of drugs, and minute anatomy, could give place to more time for the study of diagnosis," and he emphasized that in this study the special investigations must come last.

It is amusing to read what Thackrah wrote one hundred and twenty years ago. "Medical students—obliged to acquire in a couple of winters that various knowledge which ought not to be acquired in triple the time—are severely injured by the great application of mind. Hence the students who come out of the lecture room at the end of the session we should scarcely recognize as the healthy young men who entered it a few months before." Is there not still some small modicum of truth in this despite the fact that conditions for medical students have improved beyond all measure? "That various knowledge which ought not to be acquired in triple the time!"

Two recent developments are encouraging. The post-graduate courses for general practitioners—in Bristol anyhow—are of the greatest value. And the experiments in giving final-year students an insight into general practice I hope are proving successful and will be extended further.

I much admire the present system of clinical teaching and the way in which everything seems to be fitted in, but it all must mean more addition and more compression, for whereas, during the last twenty-five years, whole new subjects have been included, as far as I know nothing has been left out or even pruned. Surely the place for minutiae and detail is not in the undergraduate years at all?

And now what of the larger and more urgent problems—how to make use of this learning most effectively, and how to keep it bright? It will be a tragedy if only the lesser brains are to go into general practice, now that the old system of a more random selection no longer maintains. It is the lesser brain that will most easily atrophy in a disjoined general practice.

The most recent of the many reports on general practice—that of the Central Health Services Council has a good deal to say on the subject. In brief—general practice is, in the Committee's view, fundamental to the best practice of medicine, and to the best interests of the patient. It cannot be replaced by a congeries of specialisms, nor is it subordinate to them. Conditions of practice must be such as to attract the right type of doctor. The Committee suggests that it should be made easier for general practitioners to secure appointment in hospitals, and enter hospital specialist practice either part-time or whole-time. The Committee is of the opinion that hospitals, whenever appropriate, should plan to make beds available to general practitioners where they can retain responsibility for their patients, but where consultant advice would be available if necessary. The Committee considers that such beds would enable general practitioners to look after, for example, many confinements which now take place in hospitals solely on social grounds. "The Committee recommends the establishment of Clinical Assistantships for general practitioners in hospitals, both for their educational value, and as a training for special practice. The Committee also favour the part-time employment of suitably qualified and experienced general practitioners in all hospital grades."

This is a National Health Service report and it will be obvious that the Minister and his officials—not forgetting the Socialist Shadow Cabinet—are going to study it carefully, and will claim its authority in introducing any measures which they may think advantageous to *them*. Is there not now all the greater urgency to begin taking these evolutionary steps ourselves in order to rearticulate this over-lauded but disjoined backbone with the rest of the framework, for the good of the Body Medical and all its parts, including the bewildered "Backbone" itself; and for the good of those it has to serve?

As a result it should be possible to relieve consultants of much of their more commonplace work, to reduce gradually the registrar strength to what is compatible with the number of consultant posts available, to have each grade fully staffed, and, at the same time, to keep the ladder open and clear, as it used to be, for whoever may show the ability and resource to ascend it. What is more, a young doctor with a few hospital sessions, would be all the more acceptable as a junior partner, with his growing experience to contribute, and the supplement to his earnings.

I said a short time ago that nothing had been done. Compared with the amount of talk, and the expressions of goodwill, that is substantially correct, but it is encouraging to be able to say that, in addition to the employment of general practitioner anaesthetists in Bristol and elsewhere, there is at least one brighter exception. The Barnet Hospital Management Committee are employing six general practitioners as clinical assistants in Barnet General Hospital, two in medicine, and one each in surgery, gynaecology and obstetrics, paediatrics and, dermatology. They have also appointed twenty general practitioners to Finchley Memorial Hospital working under the appropriate consultants, and—they say—the arrangement appears to be quite satisfactory. The pertinent facts in their report are—"It is felt that, in this area, the standard of medical practice is high, and that it will be maintained, if the arrangements are continued so that most of the general practitioners may, in their turn, have an appointment of clinical assistantship. So far as the senior clinicians are concerned they work well under the scheme, and show every desire to make the best possible use of it."

"The Management Committee is strongly of the opinion that the general practitioner is so vital to the National Health Service that every effort is made to bring about a close and friendly relationship between all parties concerned. The Barnet Group Hospital Management Committee were pioneers with clinical assistantships. The Regional Board approved the arrangements and the payments involved, and gave every possible assistance in bringing the scheme into being."

I hope that there are other examples than this by now, but here is rich food for thought, and I hope that those in high places and spheres of influence will give it their serious consideration. As I have said—general practitioners have asked—but they want to be invited into the hospital service, not legislated into it. General practitioners are already considered capable of doing the work of National Service, Insurance, and Pensions Boards, and some do sessions as Industrial Medical Officers. Of these appointments, the last especially, demands special interest and ability, but who better to fill them than general practitioners who, more than any other, should have acquired an awareness of the whole man and his individual problems and difficulties?

I hope also that general practitioners may be invited, as vacancies arise, to take more part in the Local Authority Clinics. Seeing a well or ailing child or pregnant woman but hardly ever an ill one, and never attending a confinement, must be unsatisfying and sterile work, but would be an entirely different matter when done in conjunction with a daily occupation with just those things, and consequently more satisfactory to all concerned. Taken by and large, general practitioners have never asked for more than a just rate for the job, and it is generally realized that employment on sessional work would involve a comparative reduction in the permissible maximum list of patients. It therefore follows that the greater the number of sessional jobs that become available, the greater will be the number of vacancies for more general practitioners. Thus the basis of medical practice will be broadened, and general practitioners will be doing what they should be entirely capable of doing, the general work—the "ordinary work" the whole-man work, throughout the whole range of the profession, and that "Backbone" will be firmly and properly integrated in the Body Medical again.

Well now, this wavering syndrome has run its course. I have attempted to give a general practitioner's idea of the malaise from which we are all suffering, its rough and ready pathology, and the physiological methods by which it may be possible to bring about improvement—that by caring, thinking, and acting as a brotherhood we may be able to reintegrate what should be a highly cultured and finely oriented organism—the Body Medical—to our own satisfaction and the benefit of the people.

We *must not* wait upon the politicians who, with their mechanistic departmental outlook, and expediency as their yardstick, have little appreciation that for efficient function, that body requires an unworried, finely co-ordinated, integration of all its parts, which demands a strong and flexible backbone.

If, in what I have said, I may have appeared in places to be critical it has been criticism of the profession as a whole, because, in the constant race against time, we do not devote enough thought or energy to the good of the profession, from the first-year student upwards—not even to our own good—and for that we have suffered, for that we shall continue to suffer, unless and until we take corporate and undivided action about these things, instead of agreeing in groups that something ought to be done. And if in the beginning, I may have seemed to infer that I have had a strenuous life, I do not want to leave any misconception, especially in the minds of those who may be contemplating, or beginning, general practice.

It is true that I often wish for more time—to enjoy my family, to indulge my other interests, even to be a better doctor, but I have enjoyed, am still enjoying, my life immensely, and I wouldn't change my job for any other.

